# *Find Cancer Early*: Evaluation of a Community Education Campaign to Increase Awareness of Cancer Signs and Symptoms in People in Regional Western Australians

**DOI:** 10.3389/fpubh.2018.00022

**Published:** 2018-02-08

**Authors:** Emma Jane Croager, Victoria Gray, Iain Stephen Pratt, Terry Slevin, Simone Pettigrew, C. D’arcy Holman, Max Bulsara, Jon Emery

**Affiliations:** ^1^Cancer Council Western Australia, Subiaco, WA, Australia; ^2^University of Western Australia, Perth, WA, Australia; ^3^Western Australian Cancer Prevention Research Unit, Curtin University, Perth, WA, Australia; ^4^University of Notre Dame Australia, Fremantle, WA, Australia; ^5^University of Melbourne, Melbourne, VIC, Australia

**Keywords:** health promotion, early detection of cancer, community engagement, rural health, social marketing

## Abstract

**Introduction:**

Cancer outcomes for people living in rural and remote areas are worse than for those living in urban areas. Although access to and quality of cancer treatment are important determinants of outcomes, delayed presentation has been observed in rural patients.

**Methods:**

Formative research with people from rural Western Australia (WA) led to the Find Cancer Early campaign. Find Cancer Early was delivered in three regions of WA, with two other regions acting as controls. Staff delivered the campaign using a community engagement approach, including promotion in local media. Television communications were not used to minimize contamination in the control regions. The campaign evaluation was undertaken at 20 months *via* a computer-assisted telephone interview (CATI) survey comparing campaign and control regions. The primary outcome variable was knowledge of cancer signs and symptoms.

**Results:**

Recognition and recall of Find Cancer Early and symptom knowledge were higher in the campaign regions. More than a quarter of those who were aware of the campaign reported seeing the GP as a result of their exposure.

**Conclusion:**

Despite limited use of mass media, Find Cancer Early successfully improved knowledge of cancer symptoms and possibly led to changes in behavior. Social marketing campaigns using community development can raise awareness and knowledge of a health issue in the absence of television advertising.

## Introduction

There is clear evidence worldwide that cancer outcomes for people living in rural and remote regions are poorer than those living in urban areas (1). Australia is no exception, and although over the past decade much progress has been made in decreasing cancer mortality overall, there has been little progress in decreasing cancer mortality in rural and remote parts of the country (2, 3). People living in these areas are 20–30% more likely to die within 5 years of a cancer diagnosis than people from metropolitan areas (4).

Much of the research into why these disparities exist has focused on access to and quality of the treatment received by rural and remote patients (2, 5–7). As a result, policy initiatives have focused on reducing disparities in access to cancer treatment (8). Although treatment access is an important determinant of outcome, later presentation and later stage of diagnosis have also been observed in rural cancer patients (9, 10). International research indicates that the time taken to appraise symptoms and seek help (termed “patient delay”) and symptom management in primary care are key determinants of cancer outcome (11). The longer it takes to diagnose cancer, the lower the survival rate for several common cancers (12, 13).

It has recently been shown in Western Australia (WA) that rural cancer patients are less aware of the common signs and symptoms of cancer and more likely to delay seeking medical help than their urban counterparts (14, 15). There are several reasons why these delays may occur, including poor awareness of symptoms, negative beliefs about cancer outcomes, barriers to symptomatic presentation, and poor awareness of cancer risk (16–21). Low awareness of cancer symptoms and barriers to help-seeking has been shown in the UK to be associated with longer delays in help-seeking (22). Furthermore, patients’ cancer awareness and beliefs influence how promptly GPs refer them for further investigation and how promptly they receive effective treatment (22). Emery et al. have identified that core characteristics of rural Australians, such as optimism, stoicism, and machismo, probably also contribute to later presentation (15).

Social marketing involves using a range of marketing techniques and communication strategies to encourage changes in health behaviors primarily by influencing individuals’ knowledge, attitudes, and behaviors (23, 24). Although a systematic review of cancer symptom awareness campaigns published in 2009 found insufficient evidence to support the effect of social marketing campaigns on presentation to health professionals (25). More recent studies have shown effects of exposure to campaigns on presentation and earlier cancer diagnoses (26–29). Reflecting these findings, community symptom awareness campaigns to reduce late presentation have formed a major component of the UK National Awareness and Early Diagnosis Initiative (NAEDI) to improve cancer outcomes (30).

## Background and Rationale

In November 2011, Cancer Council WA launched a rural cancer symptom awareness campaign called “Find Cancer Early” in three rural regions of WA. The 2-year community-based social marketing campaign aimed to increase people’s awareness of the common signs and symptoms of breast, bowel, lung, and prostate cancers, and encourage them not to make excuses to seek help from a doctor when experiencing these signs and symptoms. The target group was people aged 40 years and above. Each region had a local campaign officer responsible for (i) establishing stakeholder relationships with community organizations and local media and (ii) the delivery of campaign messages and activities. Over the 2 years, the main campaign dissemination strategies included modest and restricted paid advertising, unpaid publicity on local radio and in local newspapers and other regional publications, and extensive community engagement. This paper reports key findings from the process and impact evaluations of the first 20 months of the Find Cancer Early campaign.

## Methods

### Campaign Theory and Development

#### The Find Cancer Early

The Find Cancer Early public awareness campaign was delivered from November 2011 to 2013 in the Goldfields (770,488 km^2^), Wheatbelt (155,256 km^2^), and Great Southern (39,007 km^2^) regions of WA. Control regions were the Midwest (470,000 km^2^), and Southwest (29,646 km^2^) regions of WA (31). The Pilbara and Kimberley regions were excluded because they were demographically different to the control and campaign regions (higher proportion of younger and Aboriginal people and transient workforces) and had other structural barriers to the early detection and treatment of cancer (e.g., greater distances to primary care and treatment centers). The campaign and control regions were matched for population size, demographics, including age, sex, Aboriginality, and socioeconomic status (as determined by postcode) and cancer incidence.

#### Find Cancer Early

Find Cancer Early campaign materials were developed based on findings from a previous exploratory mixed-methods study that identified barriers to symptom appraisal and help-seeking behavior in people from rural WA (15). Key themes identified included optimism, stoicism, machismo, fear, embarrassment, and competing demands. Concepts reflecting these themes were developed using content from Cancer Research UK’s “Spot Cancer Early” (now “Spot Cancer Sooner”) and the UK National Health Service’s “3 week cough” campaigns (32, 33), incorporating language and imagery reflecting rural Western Australian values. Concepts were tested in community fora in the campaign target regions and feedback was used to further refine the campaign materials. The resulting materials were simple, non-medical, and used “everyday” words and imagery relevant to people in rural WA. As Cancer Council WA is a familiar brand in WA, the Find Cancer Early campaign materials used the same blue and yellow color scheme. The tagline “The earlier cancer is found, the greater chance of successful treatment” was included because participants in the exploratory study wanted the campaign to focus on the positives associated with early detection, rather than the negatives associated with cancer (15). The signs and symptoms communicated were agreed on and endorsed by cancer specialists as being important for the early detection of cancer.

The primary communication was a plain-language symptom checklist, which was used in print media, posters, and banners. Other materials included four 30s radio advertisements (one for each cancer type—breast, bowel, lung, and prostate), postcards featuring rural images and quotations about symptoms relevant to the four cancers, postcards providing strategies to overcome barriers to seeking help, and a DVD featuring health professionals and rural community members discussing the common signs and symptoms of the cancers and what to do. A campaign website was also developed (www.findcancerearly.com.au). Due to concerns over contamination into control regions, the website was not actively promoted or optimized for search engines.

Five campaign officers, with a combined full-time equivalent (FTE) of three full-time workers, delivered the campaign across the three campaign regions. These campaign officers all had training or experience in health promotion or community development. They used a community engagement approach to (i) build partnerships with community organizations and local media and (ii) disseminate the campaign messages across their regions through presentations, displays, and campaign resource distribution. Paid advertising (six bursts of 2–4 weeks) and unpaid publicity on local radio and in community newspapers and other regional publications supplemented this dissemination strategy. Television and online media were not used for either paid advertising or unpaid news stories to minimize contamination of the control regions.

### Process Evaluation

Process measures included media exposure (paid and unpaid print and radio) and number of community presentations and displays delivered by campaign staff to promote the Find Cancer Early messages. Media exposure was estimated by the number and reach of paid and unpaid press and radio coverage. Newspapers and radio stations were carefully selected to minimize contamination of the control regions.

### Impact Evaluation

The primary outcome variable was knowledge of cancer signs and symptoms. Other outcome variables included campaign awareness and monitoring and acting on signs and symptoms.

Computer-assisted telephone interviews (CATI) were conducted with adults 40 years of age and older from campaign (*n* = 726) and control (*n* = 726) regions. An *a priori* power analysis determined that this sample size would be sufficient to detect a 5% difference in impact measures between campaign and control regions (34). Households were randomly selected from the Western Australian White Pages telephone directory. Participants were excluded if they were under 40, had not been resident in the region for at least 6 months, or were unable to complete the interview in English. The participation rate among those who were eligible was 94.1%.

Quotas were set to ensure equal numbers of respondents from the campaign regions and control regions and equal representation by gender and age group (40–49, 50–64, and 65+ years). Interviews were carried out during a 4-week period from July 1, 2013. Ethics approval was obtained from The University of Western Australia’s Human Research Ethics Committee (RA/4/1/4527).

To minimize order effects and priming, the questionnaire started with basic demographic information and cancer symptom knowledge before assessing campaign recall and recognition. The remainder of the questionnaire measured message salience, perceived impact, behavioral intention, and behavior change.

Symptom knowledge was assessed by one open-ended question asking respondents to nominate the most common signs and symptoms of cancer. Respondents were able to provide as many responses as desired. Responses were post-coded into categories by the interviewer.

Campaign awareness (recall plus recognition) was assessed using an existing campaign evaluation protocol (35–38). Specifically, campaign recall was evaluated by asking whether respondents had heard any health messages about cancer in the last year, and then probing for more information on those messages to identify mentions of the Find Cancer Early campaign. Campaign recognition among those exhibiting no recall was assessed by providing brief descriptions of the campaign materials and asking respondents whether they had seen them.

As per Morley et al. (37), four-point Likert-type scales (response anchors “strongly disagree” to “strongly agree”) were used to assess whether the Find Cancer Early Campaign was perceived to be believable, easy to understand, personally relevant, informative, and a cause of discomfort among those respondents exhibiting campaign recall or recognition. Overall agreement or disagreement was calculated by summing “strongly agree” with “agree” and “strongly disagree” with “disagree.” These respondents were also asked whether they had acted or considered acting on the campaign message and the nature of any such action.

### Statistical Analysis

Data were analyzed using SPSS 24, with the null hypothesis having no difference between the campaign and control regions. Pearson chi-squared tests (two-sided) were conducted on all categorical data. Continuous data were analyzed using an independent-sample’s *t*-test.

## Results

### Sample Characteristics

Sample characteristics are shown in Table 1. Quota sampling ensured that the campaign and control samples did not differ on key demographic characteristics (sex and age). There was no difference observed between the campaign and control samples in the proportion of respondents reporting a previous cancer diagnosis (15.4 vs 16.7%, respectively, *p* = 0.52).

**Table 1:** Campaign and control sample comparison.

Sample characteristic		Campaign		Control		Campaign vs control
		*n*	%	*n*	%	
Sex	Male	356	49.0	354	48.7	χ^2^(1, *N* = 1,452) = 0.01, *p* = 0.92
	Female	370	51.0	372	51.3	

Age	40–49	231	31.8	222	30.6	χ^2^(2, *N* = 1,452) = 0.26, *p* = 0.88
	50–64	248	34.2	252	34.7	
	65+	247	34.0	252	34.7	

History of cancer	Yes	121	16.7^c^	112	15.4	χ^2^(1, *N* = 1,452) = 0.414, *p* = 0.52
	No	605	83.3^c^	614	84.6	

Total		726	100	726	100	

### Process Evaluation

After 20 months, 214,317 Find Cancer Early publications had been distributed, 435 community sessions were delivered, and 691 unpaid media items had been achieved. This was in addition to paid advertising in local print and radio (Table 2).

**Table 2:** Process measures November 2011–July 2013.

Measure	Number	Estimated reach
Checklists distributed	130,388	130,388
Postcards distributed	78,317	78,317
DVDs distributed	4,170	4,170
Aboriginal resources distributed	1,262	1,262
Community presentations	312	6,549
Static displays	123	
Regional newspaper articles	89	573,380
Local newspaper/newsletter articles	602	283,520
Radio interviews	10	
Paid newspaper advertisements (1/4 page)	162	2,262,498
Paid radio advertisements (30s)	1,490	

Total population aged 40 years and above	121,600	

### Campaign Impact on Knowledge

During the telephone interview, knowledge of cancer signs and symptoms was assessed prior to campaign awareness to minimize priming. Participants were asked: “Can you please tell me what you think are the most common signs and symptoms of cancer?” This question was based on Cancer Research UK’s Cancer Awareness Measure (CAM) for symptom awareness (39). The term “signs and symptoms” was explained if needed, but no further prompting occurred. The total number of cancer signs and symptoms correctly identified was higher in the campaign regions than in the control regions (1.65 vs 1.31, *p* = 0.000). Notably, people in the campaign regions were more likely to identify “blood in your poo,” “blood in your pee,” and “an unusual pain, lump, or swelling” than people in the control regions (Table 3).

**Table 3:** Knowledge of cancer signs and symptoms.

Symptom recall	Campaign		Control		Campaign vs control	
	*n*	%	*N*	%	χ^2^ (1, *N* = 1,452)	*p*
Coughing up blood (included once)	56	7.7	51	7.0	0.25	0.616
A cough or croaky voice	53	7.3	38	5.2	2.64	0.104
Becoming more short of breath	39	5.4	40	5.5	0.01	0.908
Blood in your pee (included once)	128	17.6	60	8.3	28.25	0.000
Blood in your poo	227	31.3	136	18.7	30.42	0.000
Problems peeing	29	4.0	22	3.0	1.00	0.318
Looser poo (diarrhea)	44	6.1	40	5.5	0.20	0.653
An unusual pain, lump, or swelling	464	63.9	425	58.5	4.41	0.036
Unexplained weight loss	157	21.6	140	19.3	2.80	0.094

### Campaign Awareness

Residents of the campaign regions were more likely than residents of the control region to have “heard or seen any health message about cancer in the last year,” (88.2 vs 80.3%; χ^2^[1, *N* = 1,452] = 16.84, *p* = 0.000). Residents of the campaign regions had greater recall and recognition of the Find Cancer Early campaign (Table 4). Total awareness (prompted and unprompted) was 61.4% in the campaign regions vs 20.4% in the control regions (χ^2^[1, *N* = 1,452] = 253.00, *p* = 0.000).

**Table 4:** Recall and recognition of the *Find Cancer Early* campaign.

Campaign awareness	Campaign		Control	
	*N*	%	*n*	%
Unprompted recall	56	7.7	12	1.7
Prompted recognition	390	53.7	136	18.7
Not aware	280	38.6	578	79.6
Total	726	100	726	100

*Campaign vs control: χ^2^(2, N = 1,452) = 254.63, p = 0.000*.

### Campaign Salience

Most people who were aware of the campaign reported that it was easy to understand (98.5%), believable (98.8%), relevant to them (75.4%), and did not make them feel uncomfortable (82.6%). However, only 44% of people who were aware of the campaign felt that it taught them something new.

### Campaign Impact on Intention and Behavior

Among those who were aware of the campaign, almost half (46.9%) thought about doing something, and more than a third (34.3%) reported doing something as a result of exposure. As shown in Table 5, the most common intention and behavior was to see a doctor/GP.

**Table 5:** Intentions and behaviors among those aware of the *Find Cancer Early* campaign.

Campaign impact	Aware of the campaign (*n* = 405)	
**Intention (thought about…)**	***n***	**%**
Seeing a doctor/GP	117	28.9
Monitoring symptoms	43	10.6
Increasing symptom knowledge	15	3.7
**Behavior**		
Saw a doctor/GP	105	25.9
Monitored symptoms	22	5.4

## Discussion

Awareness of the importance of the early detection of cancer, knowledge of cancer signs and symptoms, and self-reported cancer-related intentions or behaviors were higher in the Find Cancer Early campaign regions than in the control regions. Although the quasi-experimental, post-test design limits causal inference, the large effect sizes and lack of plausible confounders (resulting from demographically matched campaign and control regions and low likelihood of pre-existing differences in outcome measures) suggest that Find Cancer Early was a successful campaign. Outcomes were achieved despite the inability to use television and online media because of the need to avoid contamination in the control regions. Importantly, the results suggest that it is possible to develop a successful social marketing campaign without these media forms when the target audience is rural communities and effort is invested in community development strategies. Further, the results support the potential effectiveness of the alternative social marketing activities used in the intervention, including community engagement by the regional campaign officers and delivery of the campaign through community partnerships and local paid media. A similar approach has previously been reported for successful delivery of the Act-Belong-Commit mental health campaign (40).

The encouraging outcome of the Find Cancer Early campaign in raising awareness is likely to be the result of two primary factors. First, extensive formative research was carried out with people living in regional WA to guide the development and refinement of campaign messages and materials (15). This resulted in the implementation of positively framed campaign messages using simple language and a clear call to action that was salient for the intended target audience. Second, the community engagement approach used alongside the limited purchased media was important in enhancing the reach of the campaign. Campaign officers successfully formed ongoing partnerships throughout their regions to promote campaign messages. Importantly, the media buy was supplemented substantially with unpaid media coverage that was generated by campaign officers developing effective working relationships with local community groups and media outlets. This was achieved by building personal relationships with local editorial staff and providing story and picture opportunities relevant to the communities targeted by these regional publications.

Due to the restriction on TV and online advertising, this study could not test the relative impact of mass media advertising in communicating early detection messages compared to the community-based approach adopted in the Find Cancer Early campaign. It is likely that greater investment in the intervention and the use of high impact media channels such as television would have achieved better outcomes. Future campaign evaluation will potentially allow for these comparisons to be made.

Symptom awareness campaigns remain a major strategy internationally as part of cancer control. For example, they have formed a major component of the UK NAEDI to improve cancer outcomes (30). Initial observational data from a lung cancer awareness campaign in England showed greater intentions to attend a GP with a cough, increases in chest X-ray requests, and higher incidence of lung cancer diagnoses (26).

This evaluation has limitations. Most notable, as previously mentioned, the cross-sectional, single-time-point data collection does not allow for causation to be inferred. In addition, individual-level socioeconomic data were not collect, so it is not possible to say that the control and campaign samples were matched or representative for education or income.

There are lessons for practice that can be taken from this evaluation. First, the importance of formative research in the development of the campaign materials. It is likely that the strong campaign awareness was in part due to the salience of the materials. Second, community engagement—or on the ground health promotion—remains an effective yet often overlooked strategy in changing knowledge, attitude, and beliefs, at least in older adults living in regional communities. Finally, it is important to evaluate behavior change campaigns, particularly when delivered *via* non-traditional methods.

## Conclusion

Evaluation of Find Cancer Early demonstrates the potential effectiveness in rural communities of a modest social marketing campaign combined with community development strategies in increasing awareness of cancer signs and symptoms.

## Author Contributions

TS, JE, CH, VG, and EC conceptualized and designed the study. EC managed campaign and staff. VG developed and tested materials, and coordinated campaign delivery. IP developed and tested evaluation survey and analyzed data. MB provided statistical expertise to ensure sample size powered adequately. SP contributed to evaluation survey design and analysis. JE and CH conceptualized and designed the Improving Rural Cancer Outcomes (IRCO) study. All authors have been involved in drafting and critical evaluation of the manuscript.

## Conflict of Interest Statement

The authors declare that the research was conducted in the absence of any commercial or financial relationships that could be construed as a potential conflict of interest.
